# LPA_3_ Receptor Phosphorylation Sites: Roles in Signaling and Internalization

**DOI:** 10.3390/ijms25105508

**Published:** 2024-05-18

**Authors:** K. Helivier Solís, M. Teresa Romero-Ávila, Ruth Rincón-Heredia, J. Adolfo García-Sáinz

**Affiliations:** 1 Departamento de Biología Celular y Desarrollo, Instituto de Fisiología Celular, Universidad Nacional Autónoma de México, Ciudad Universitaria, Ap. Postal 70-600, Ciudad de México 04510, Mexico; samsonyte09@gmail.com (K.H.S.); tromero@ifc.unam.mx (M.T.R.-Á.); 2 Unidad de Imagenología, Instituto de Fisiología Celular, Universidad Nacional Autónoma de México, Ciudad Universitaria, Ap. Postal 70-600, Ciudad de México 04510, Mexico; rrincon@ifc.unam.mx

**Keywords:** Lysophosphatidic acid: LPA, Lysophosphatidic acid receptor 3, LPA_3_, phosphorylation sites, signaling

## Abstract

Lysophosphatidic acid (LPA) type 3 (LPA_3_) receptor mutants were generated in which the sites detected phosphorylated were substituted by non-phosphorylatable amino acids. Substitutions were made in the intracellular loop 3 (IL3 mutant), the carboxyl terminus (Ctail), and both domains (IL3/Ctail). The wild-type (WT) receptor and the mutants were expressed in T-REx HEK293 cells, and the consequences of the substitutions were analyzed employing different functional parameters. Agonist- and LPA-mediated receptor phosphorylation was diminished in the IL3 and Ctail mutants and essentially abolished in the IL3/Ctail mutant, confirming that the main phosphorylation sites are present in both domains and their role in receptor phosphorylation eliminated by substitution and distributed in both domains. The WT and mutant receptors increased intracellular calcium and ERK 1/2 phosphorylation in response to LPA and PMA. The agonist, Ki16425, diminished baseline intracellular calcium, which suggests some receptor endogenous activity. Similarly, baseline ERK1/2 phosphorylation was diminished by Ki16425. An increase in baseline ERK phosphorylation was detected in the IL3/Ctail mutant. LPA and PMA-induced receptor interaction with β-arrestin 2 and LPA_3_ internalization were severely diminished in cells expressing the mutants. Mutant-expressing cells also exhibit increased baseline proliferation and response to different stimuli, which were inhibited by the antagonist Ki16425, suggesting a role of LPA receptors in this process. Migration in response to different attractants was markedly increased in the Ctail mutant, which the Ki16425 antagonist also attenuated. Our data experimentally show that receptor phosphorylation in the distinct domains is relevant for LPA_3_ receptor function

## 1. Introduction

Lysophosphatidic acid (LPA) participates in lipid metabolism, and it is also known to induce a plethora of effects acting as a messenger in intercellular communication, i.e., it is a “bioactive lipid”, exerting those actions through interaction with different receptors, mainly those of the G protein-coupled receptor superfamily [1,2,3]. Six of these receptors are generally considered to constitute the LPA receptor subfamily (LPA_1–6_). These receptors are expressed almost ubiquitously and frequently exhibit promiscuous interaction with various G proteins that activate many signaling pathways [1,2,3].

The LPA_3_ receptor, the subject of this work, has a vast expression in tissues, and it is particularly abundant in the heart, testicles, prostate, and pancreas, while it has lower expression in the lungs, brain, colon, duodenum, stomach, thyroid, and the gallbladder [2]. This receptor participates in many physiological functions and pathological processes, including chemotaxis [4], migration [5], proliferation [6], differentiation [7], embryo implantations [8], and determination of vertebrate left-right patterning during embryogenesis [9], among many others. The relevant role of LPA and its receptors in cancer has been recently reviewed (see [10] and references therein). In ovarian cancer, LPA_3_ expression is considered a therapeutic target and a poor prognosis marker [6,11].

As expected for a G protein-coupled receptor, LPA_3_ presents in its structure seven transmembrane domains, connected by three intracellular loops and three extracellular loops, an extracellular amino terminus, and an intracellular carboxyl terminus (Ctail). This receptor interacts mainly with two different G protein types, G_αi/o_ and G_αq/11_, triggering different signaling processes via their α and βγ subunits (reviewed in [1,2,3,12,13]). It is known that agonist activation increases calcium signaling, activation of protein kinase C (PKC), phosphoinositide 3-kinase, and the MAP kinase cascade, among other pathways (see [14] and submitted manuscript [15]). 

It is generally accepted that GPCR agonist activation promotes a series of conformational changes that expose sites that can be phosphorylated by a variety of protein kinases, including but not limited to G protein-coupled receptor kinases and second messengers-activated kinases, such as protein kinase A and PKC (reviewed in [16,17,18,19]). Such phosphorylation seems critical in defining interaction with the essential proteins, β-arrestins, which participate in receptor desensitization and internalization [20,21,22]. It is worth mentioning that the specific sites phosphorylated in a receptor, rather than the overall state of phosphorylation, is what is functionally relevant. There is evidence that a given receptor can be phosphorylated at distinct sites, and it has been proposed that such difference can lead to distinct receptor functional outcomes, i.e., distinct routes of internalization, subcellular destinations, and different final actions. These ideas are known as the “phosphorylation barcode hypothesis” (reviewed in [17,18,19,23,24,25]).

LPA_3_ receptors activation, expressed in Flp-In T-Rex HEK293 cells, increases intracellular calcium, ERK 1/2 phosphorylation, induces LPA_3_ phosphorylation, favors receptor interaction with β-arrestin-2 and receptor internalization (submitted manuscript [15]). Similarly, the PKC activator, phorbol myristate acetate (PMA), induced LPA_3_ receptor phosphorylation, interaction with β-arrestin-2, receptor desensitization, and internalization. In addition, we immunopurified the LPA_3_ receptor and detected, using mass spectrometry, the sites phosphorylated in cellulo under baseline and stimulated conditions. Phosphorylated residues were detected in the intracellular loop 3 (IL3) (S221, T224, S225, and S229) and in the carboxyl terminus (Ctail) (S321, S325, S331, T333, S335, Y337, and S343) (submitted manuscript [15]). In the present work, the function of the wild-type (WT) LPA_3_-GFP receptor construct was compared to mutants in which the sites detected phosphorylated in the IL3, Ctail, or both domains (IL3/Ctail) were substituted by non-phosphorylatable amino acids (i.e., serine-alanine and threonine-valine substitutions). A cartoon showing the distinct LPA_3_ receptor mutants is presented in Supplementary Figure S1. These constructs allowed us to observe that the sites in these domains play different roles in LPA_3_ receptor signaling and regulation.

## 2. Results

**Figure 1:**
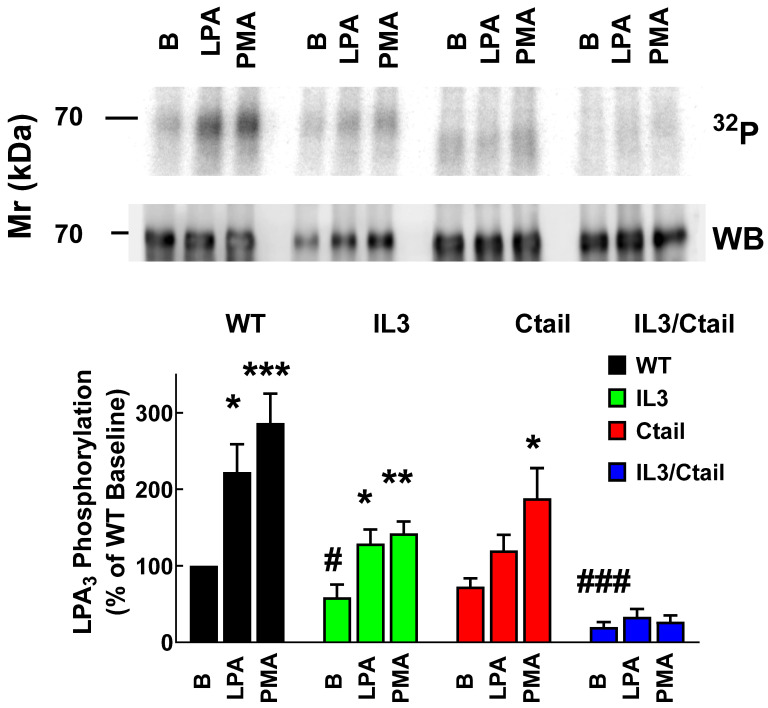
LPA- and PMA-induced LPA_3_ receptor phosphorylation. Cells expressing the WT and mutant receptors were challenged with 1 µM LPA or 1 µM PMA for 15 min. Receptor phosphorylation is expressed as the percentage of the WT baseline value. The means are plotted, and vertical lines indicate the SEM of 7–9 experiments performed on different days using different cell cultures. *** *p* < 0.001, ** *p* < 0.01, * *p* < 0.05 vs. baseline value of each mutant; ### *p* < 0.001, # *p* < 0.05 vs. WT baseline (B) representative autoradiographs (^32^P) and Western blots (WB) are presented above the graph. Representative autoradiographs (^32^P) and Western blots (WB) are presented above the graph.

Receptor phosphorylation experiments were performed in cells expressing the WT or the mutant LPA_3_ receptors. All these experiments were run in parallel and in the same gels and membranes to allow a proper comparison; cells were incubated without any agent or presence of 1 µM LPA or 1 µM PMA for 15 min (optimal time and agent concentrations). As shown in Figure 1, in cells expressing the WT receptor, a light but well-defined baseline receptor phosphorylation was observed, which was markedly increased (two to three-fold) by incubation with the agonist, LPA, or the PKC activator, PMA, in agreement with previous results [26]. Baseline phosphorylation was hardly detectable in cells expressing the IL3/Ctail receptor mutant and no effect of LPA or PMA was observed (Figure 1). In cells expressing the IL3 receptor mutant, baseline phosphorylation was decreased (≈50%) when compared to those expressing the WT, and the effects of LPA and PMA were observed but decreased (Figure 1). In cells expressing the Ctail mutant, baseline receptor phosphorylation was slightly decreased (≈30%; statistically insignificant) as compared to that in the WT-expressing cells; LPA induced a statistically insignificant increase, and PMA only marginally increased receptor phosphorylation (Figure 1). It is worth mentioning that, as shown in Figure 1 (representative image, samples were run in the same gel) receptor migration was slightly faster in cells expressing the Ctail and IL3/Ctail mutants. The reason for this is unclear; the plasmids employed were sequenced again and found to be correct. 

**Figure 2:**
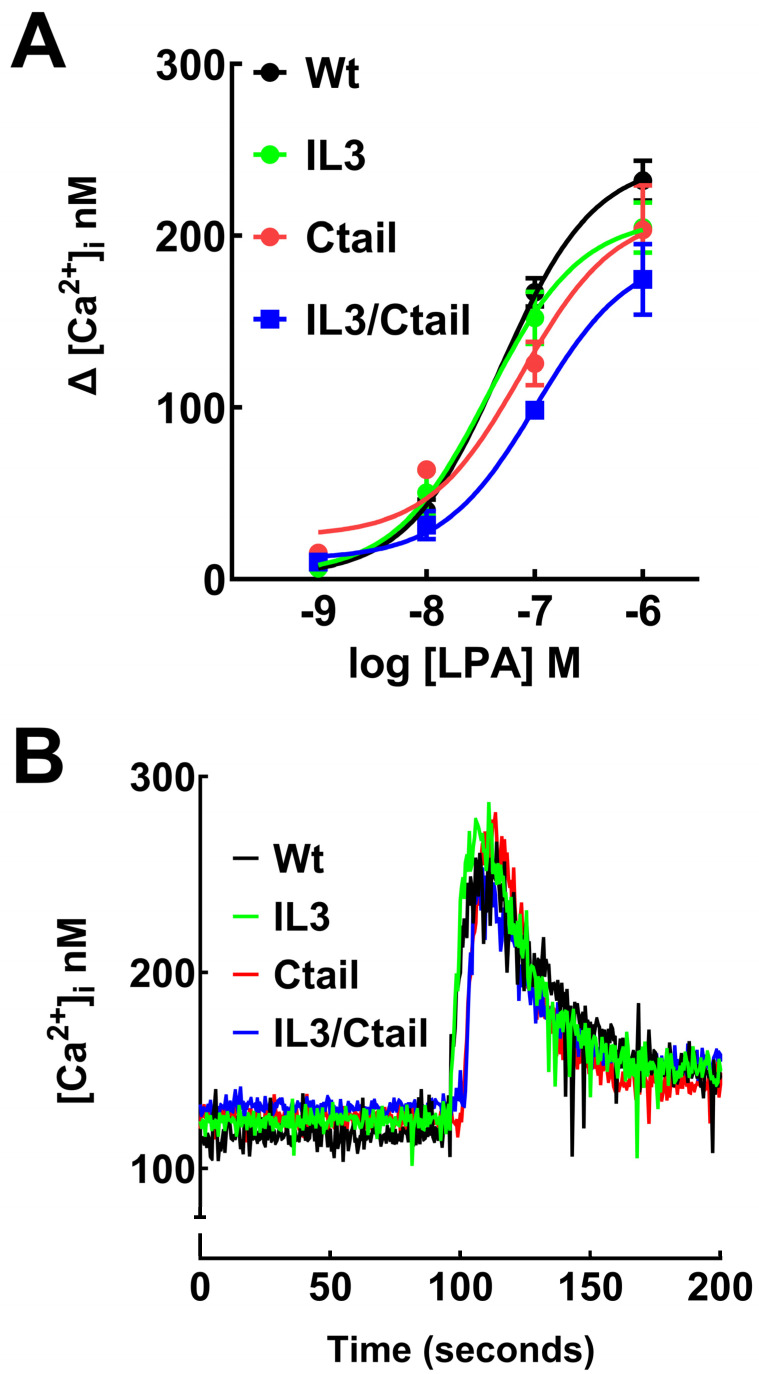
Increases in intracellular calcium in cells expressing WT or mutant LPA_3_ receptors. Panel (**A**), concentration–response curves in observed cells expressing the different LPA_3_ receptors. The abscissa indicates the increments in calcium concentration. The means are plotted, and vertical lines indicate the SEM of 5 experiments performed on different days and distinct cell cultures. Panel (**B**); cells expressing the WT and mutant receptors were challenged with 1 µM LPA. Representative calcium tracings are shown.

LPA was able to induce an increase in intracellular calcium concentration in cells expressing the WT and mutant LPA_3_ receptors (Figure 2). The response observed with 1 µM LPA was very similar in cells expressing the distinct receptors in the speed of the increases, the maxima reached, and the return toward baseline values, as shown in the representative calcium tracings (Figure 2B). However, in the concentration–response curves, a slight shift to the right was noticed, particularly in the IL3/Ctail mutant that did not reach an evident saturation at 1 µM LPA (Figure 2A). Nevertheless, the changes were relatively small. It is worth mentioning that the LPA inhibitor, Ki16425, decreased the baseline intracellular calcium concentration in cells expressing the distinct LPA_3_ receptor constructs (Supplementary Figure S2), the effect being more noticeable in cells expressing the Ctail mutant. These intracellular calcium concentration diminutions were consistently observed and statistically significant.

**Figure 3:**
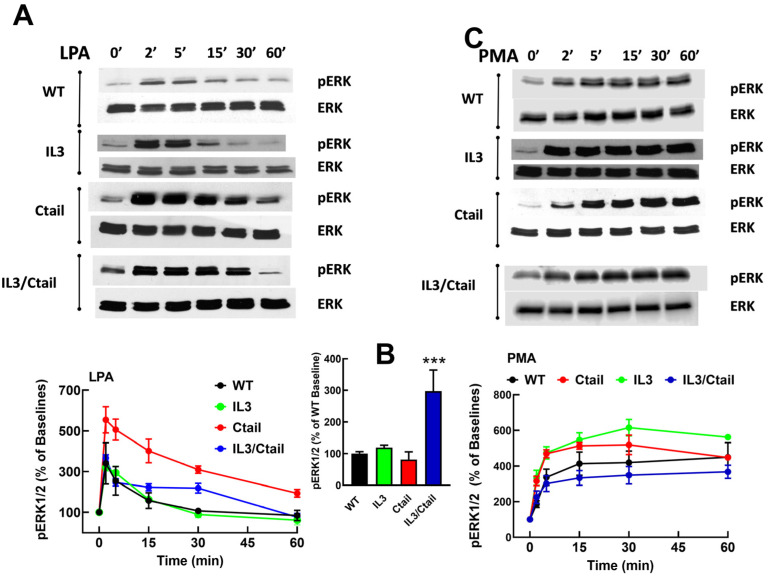
Time-course of LPA- and PMA-induced ERK 1/2 phosphorylation in cells expressing WT or mutant LPA_3_ receptors. Cells were stimulated with 1 µM LPA (Panel (**A**)) or 1 µM PMA (Panel (**C**)). Data are presented as the percentage of the baseline observed in the same cells. Panel (**B**) shows the comparative baseline values normalized to that of cells expressing the WT receptor (100%). The mean is plotted, and vertical lines indicate the SEM of 5 experiments performed on different days using distinct cell cultures. *** *p* < 0.0001 vs. WT baseline value. Representative Western blots are presented above the figure.

We previously observed that in cells expressing the LPA_3_ receptor, LPA and PMA increase ERK 1/2 phosphorylation [26]. When the effects of LPA and PMA were tested in cells expressing the WT and mutant receptors, similar patterns of ERK phosphorylation were observed (Figure 3). However, some differences were noticed. For example, the baseline ERK 1/2 phosphorylation state was noticeable but relatively small in WT receptor-expressing cells; however, in cells expressing the IL3/Ctail mutant, the baseline signal was consistently more intense (Figure 3B, see also representative immunoblots in Figure 3). In Figure 3A,C, the effects of LPA and PMA were calculated employing the baselines of cells expressing each receptor studied as a reference (100%). We considered the possibility that the decreased LPA- and PMA-induced responses observed with cells expressing the IL3/Ctail mutant receptor could only be just apparent (i.e., due to the high baseline value).

Similarly, an increase in the LPA- and PMA-mediated ERK 1/2 phosphorylation was observed in cells expressing the Ctail mutant, but baseline ERK 1/2 phosphorylation in cells expressing this mutant was variable and relatively faint in most experiments. Despite these differences, the general pattern of the responses was similar in cells expressing the distinct LPA_3_ mutants. Experiments were performed simultaneously with cells expressing the distinct LPA_3_ receptors (baseline and stimulations for 2 and 60 min), and samples were also run in the same gels; in these experiments, the baseline obtained with the WT-expressing cells was considered 100%. These data confirmed that the baseline signal was more prominent in the IL3/Ctail mutant (*p* < 0.001) than in cells expressing the WT receptor. The data also evidenced LPA’s rapid and transient effect in the different mutants and the slower but more sustained action of PMA (Supplementary Figure S3). The general patterns of the responses were similar in cells expressing the distinct LPA_3_ receptors. In cells expressing the distinct LPA_3_ receptor constructs, Ki16425 diminished ERK 1/2 phosphorylation under baseline conditions and when stimulated by 100 nM LPA, but not when 100 ng/mL EGF was the stimulus (Supplementary Figure S4).

**Figure 4:**
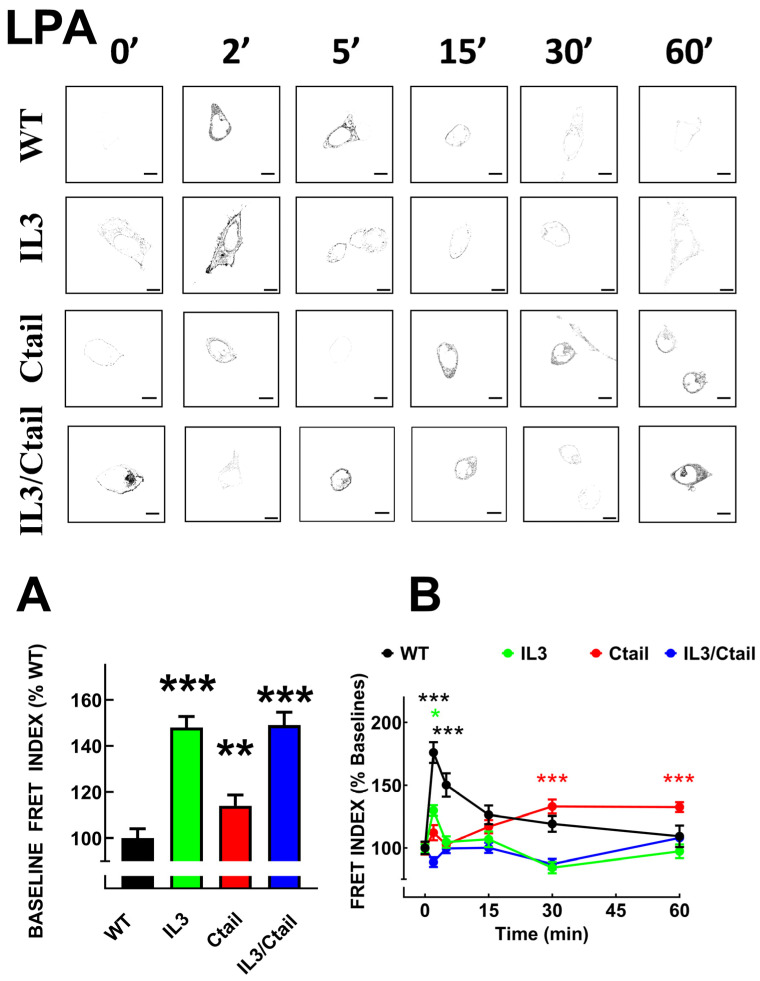
Time-course of LPA-induced LPA_3_-β-arrestin 2 interaction in cells expressing WT or mutant receptors. Panel (**A**) baseline FRET index observed in cells expressing the distinct LPA_3_ receptors. The baseline WT FRET index was considered as 100%. Panel (**B**) Cells were incubated for the times indicated with 1 µM LPA. Data are presented as the percentage of the baseline observed with cells expressing each receptor. The means are plotted, and vertical lines indicate the SEM of 7–8 experiments performed on different days and cell cultures; 7–8 images were analyzed for each condition in each experiment. In Panel (**A**), *** *p* < 0.001 and ** *p* < 0.01 vs. WT value; in Panel (**B**), *** *p* < 0.001 and * *p* < 0.05 vs. respective baseline FRET value. Representative FRET index images are presented above the graph. Bars 10 µm.

**Figure 5:**
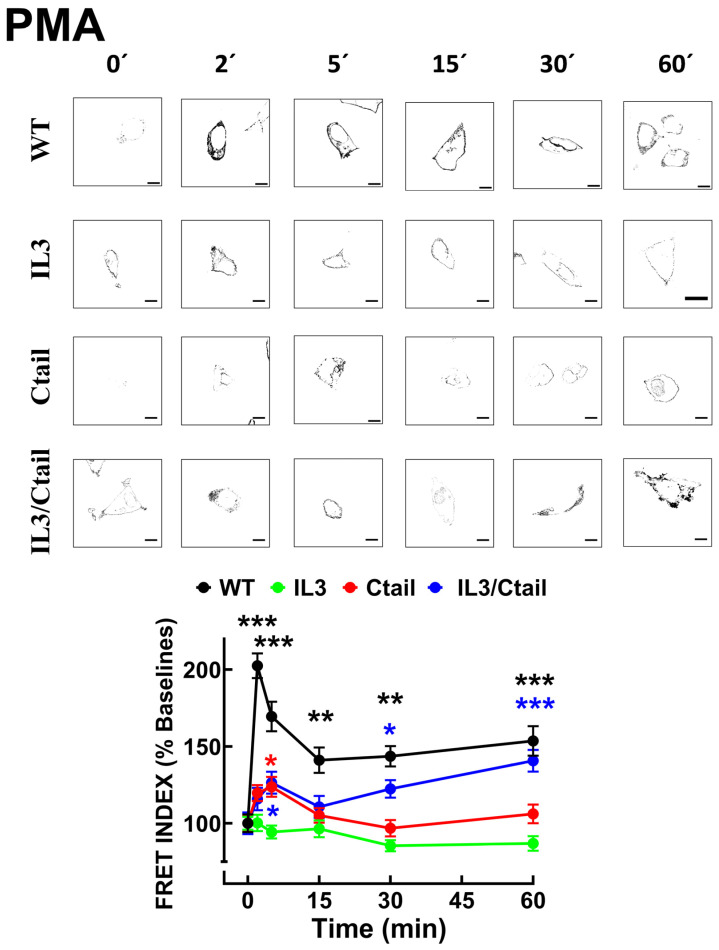
Time-course of PMA-induced LPA_3_-β-arrestin 2 interaction in cells expressing WT or mutant receptors. Cells were incubated for the times indicated with 1 µM PMA. Data are presented as the percentage of the baseline observed with cells expressing each receptor. The means are plotted, and vertical lines indicate the SEM of 7–8 experiments performed on different days and cell cultures; 7–8 images were analyzed for each condition in each experiment. *** *p* < 0.001, ** *p* < 0.01, and * *p* < 0.05 vs. respective baseline FRET value. Representative FRET index images are presented above the graph. (Bars 10 µm).

As observed with many G protein-coupled receptors, LPA_3_ agonist activation induces rapid recruitment of the β-arrestin 2, as evidenced by colocalization and FRET (submitted manuscript [15]). We analyzed LPA_3_ receptor-β-arrestin 2 interaction using FRET in cells expressing the distinct receptor mutants. As shown in Figure 4 and Figure 5, the baseline FRET index signal was low in cells expressing the WT receptor. Baseline FRET was slightly increased in cells expressing the Ctail mutant, whereas cells expressing receptors with the IL3 substitutions (i.e., the IL3 and the IL3/Ctail mutants) exhibited a more prominent baseline signal (Figure 4A and representative images in Figure 4 and Figure 5). In agreement with our previous observation, 1 µM LPA rapidly (maximum at 2–5 min) increased the LPA_3_-β-arrestin 2 FRET signal that decreased progressively at more extended times of incubation (Figure 4). In cells expressing the IL3 receptor, a small signal was observed at 2 min of stimulation and decreased afterward. Ctail mutant-expressing cells showed an insignificant signal increase at 2 min of incubation with LPA but a more robust response at 30 and 60 min; i.e., a delayed but more sustained effect was detected in cells expressing the Ctail mutant; nevertheless, it was smaller than that observed in WT-expressing cells (Figure 4). Cell expressing the IL3/Ctail mutant maintained the increased signal observed at the baseline in the presence of 1 µM LPA, and only a faint increase was detected at 60 min (Figure 4).

In cells expressing the WT receptor, PMA also rapidly increased LPA_3_-β-arrestin interaction, as indicated by the FRET index (Figure 5); a decrease was observed after this initial signal peak. No apparent increase in receptor-β-arrestin interaction was observed in response to PMA in cells expressing the IL3 mutant. In cells expressing the Ctail mutant, a slight and brief increase (2 and 5 min) in FRET was observed which decreased afterward. In cells expressing the IL3/Ctail mutant, a similar slight and brief increase in FRET signal was observed, but in this case, it was followed by further increases at 30 and 60 min, reaching at the latter time a similar value than that observed with cells expressing the WT receptor, with the same treatment (Figure 5).

**Figure 6:**
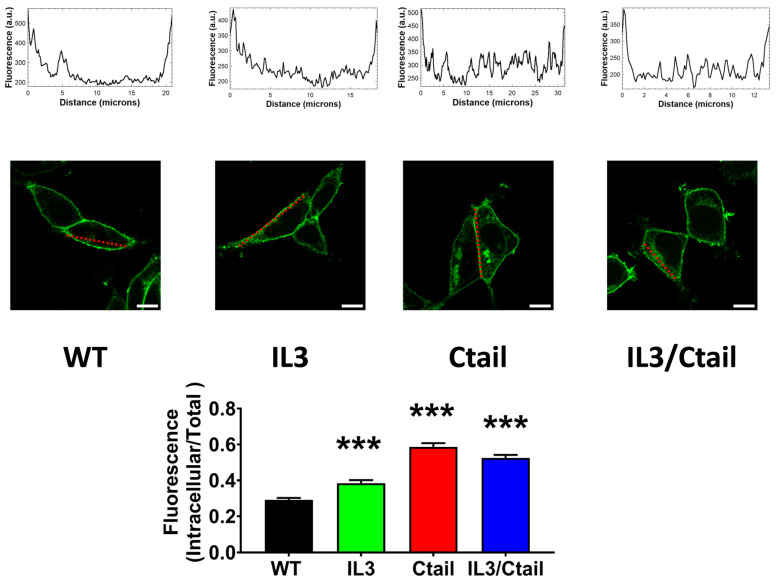
Baseline intracellular fluorescence of cells expressing WT and mutant LPA_3_ receptors. Cells expressing the different LPA_3_ receptor constructs were analyzed before any stimulation. Total fluorescence and that not located in the plasma membrane region were quantified, and the ratio was obtained. The means are plotted, and vertical lines indicate the SEM of 7–8 experiments performed on different days and cell cultures; 7–8 images were analyzed (i.e., 49 to 64). *** *p* < 0.001 vs. WT. Representative images are presented above the graph (Bars 10 µm); a red line indicates the region in which the fluorescence scan was performed (notice that the large nucleus was avoided (see also [15])). The fluorescence scans are presented above the cell images.

We next examined the baseline and stimulated localization of the WT receptor and the distinct receptor mutants. As shown in Figure 6, the cells expressing all these receptors showed marked fluorescence, patently delineating the plasma membrane; however, some differences were noticed. In cells expressing the WT LPA_3_ receptors, only a fraction (≈25%) of the cell’s fluorescence was located intracellularly. We scanned the cells (indicated with a red line in the images), and fluorescence profiles are indicated in the diagrams above the micrographs; as it can be observed, most fluorescence is present in the borders (plasma membrane) of the scan along the WT-expressing cell; some fluorescence in the cytoplasm was also noticed and evidenced as tiny peaks in the scan. It is worth mentioning that the large nucleus of these cells (see also [15]) makes it challenging to select the region to be scanned. In cells expressing the IL3 receptor mutant, intracellular fluorescence increased (>30% of the total fluorescence detected), and this was evidenced in both the photograph and the scan (Figure 6). The intracellular presence of fluorescence was even more intense in cells expressing the Ctail and IL3/Ctail mutants, reaching ≈50–60% of the total (Figure 6).

**Figure 7:**
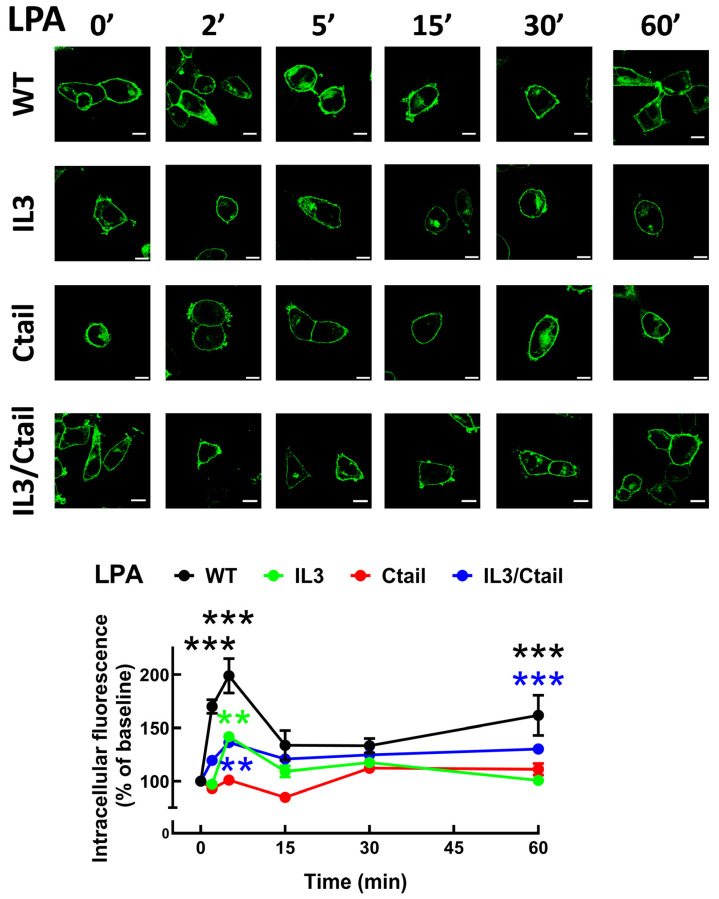
LPA-induced LPA_3_ receptor internalization. Cells expressing the different LPA*_3_* receptor constructs were stimulated with 1 µM LPA, and intracellular fluorescence was quantified; data were normalized to the baseline obtained for each receptor construct. The means are plotted, and vertical lines indicate the SEM of 7–8 experiments performed on different days and cell cultures; 7–8 images were analyzed (i.e., 49 to 64). *** *p* < 0.001 and ** *p* < 0.01 vs. respective baselines. Representative images are presented above the graph (Bars 10 µm).

**Figure 8:**
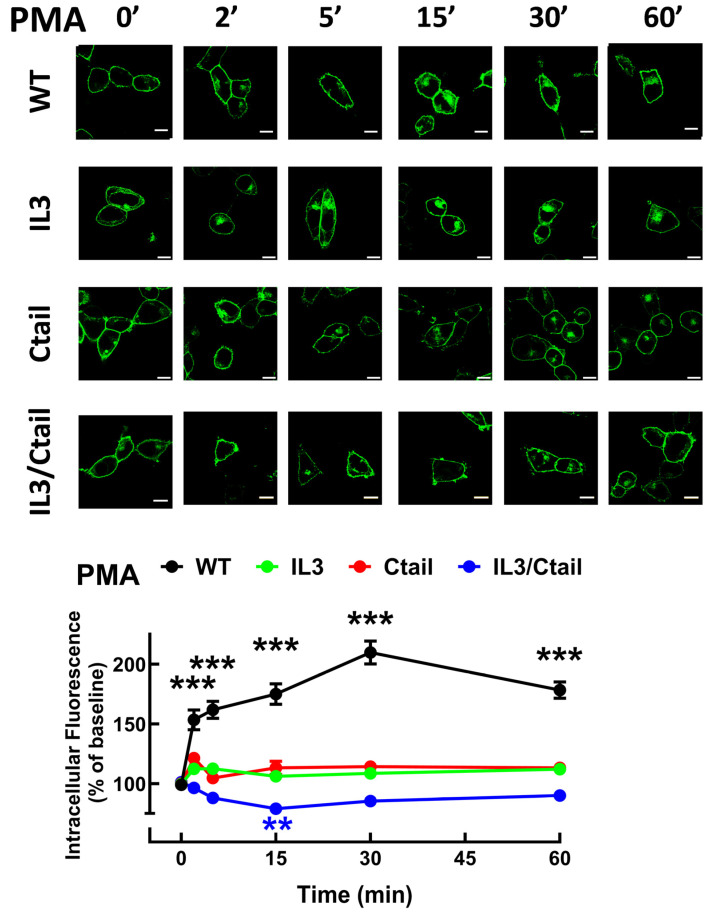
PMA-induced LPA_3_ receptor internalization. Cells expressing the different LPA_3_ receptor constructs were stimulated with 1 µM PMA, and intracellular fluorescence was quantified; data were normalized to the baseline obtained for each receptor construct. The means are plotted, and vertical lines indicate the SEM of 7–8 experiments performed on different days and cell cultures; 7–8 images were analyzed (i.e., 49 to 64). *** *p* < 0.001 and ** *p* < 0.01 vs. respective baseline value. Representative images are presented above the graph (Bars 10 µm).

In cells expressing the LPA_3_ WT receptor, 1 µM LPA induced a rapid receptor internalization (maximal at 2–5 min), which decreased at 15 min and increased again at 60 min; it was maintained above the initial level through the experiment (60 min) (Figure 7). Cells expressing the IL3 and IL3/Ctail mutants showed minimal increases in intracellular fluorescence at early times that were maintained throughout the incubation. No LPA-induced change in intracellular fluorescence was observed in cells expressing the Ctail receptors. When cells expressing the WT receptor were challenged with 1 µM PMA, rapid receptor internalization was observed that slightly further increased at 30 min and was above baseline during the whole incubation period (Figure 8). No PMA-induced internalization was observed in cells expressing the IL3 or Ctail mutants and, unexpectedly, in cells expressing the IL3/Ctail mutant a slight decrease in intracellular fluorescence was observed (Figure 8). 

**Figure 9:**
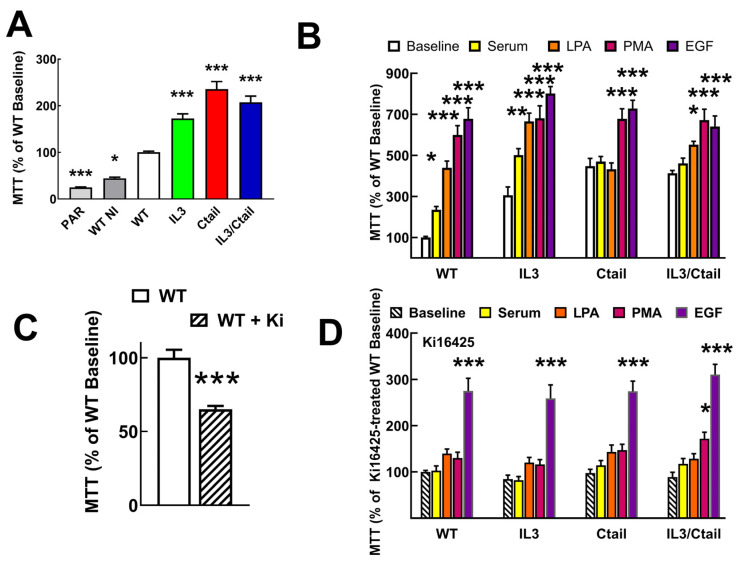
Proliferation of cells expressing the different LPA_3_ receptor constructs. Panel (**A**) cell proliferation was determined using the MTT assay indicated in Section 4. Panel (**A**). Baseline proliferation observed in Flp-In T-Rex HEK293 cells (PAR), cells transfected with the WT plasmid (but not induced; WT NI), and cells expressing the different LPA_3_ receptor constructs. Data were normalized to the value obtained with cells expressing the WT receptor (100%). The means are plotted, and vertical lines indicate the SEM of 12 experiments in duplicate performed on different days and cell cultures. * *p* < 0.05, *** *p* < 0.001 vs. WT baseline. Panel (**B**) cells expressing the different LPA_3_ receptor constructs were challenged with no agent (Baseline), 10% serum, 1 µM LPA, 1 µM PMA, or 100 ng/mL EGF. Data were normalized to the baseline obtained with cells expressing the WT receptor (100%). The means are plotted, and vertical lines indicate the SEM of 8 experiments in duplicate performed on different days and cell cultures. * *p* < 0.05, ** *p* < 0.01, and *** *p* < 0.001 vs. the baseline or each cell line. Panel (**C**) baseline proliferation in cells expressing the WT receptor incubated in the absence (100%) or presence of 1 µM Ki16425. The means are plotted, and vertical lines indicate the SEM of 8 experiments performed in duplicate on different days and cell cultures. *** *p* < 0.001 vs. absence of inhibitor. Panel (**D**) experiments were performed as indicated for Panel (**B**), but all samples contained 1 µM Ki16425. Other indications as in Panel (**B**).

Proliferation and migration, two long-term LPA actions, were also studied, considering the roles of the LPA_3_ receptor in cancer. It was also observed that cells expressing the mutant receptors reach confluence in the Petri dishes more rapidly than those expressing the WT (Figure 9A). We studied receptor proliferation using the MTT assay [27], which determines the oxidative capacity of the culture, an index of cell number. 

Both parenteral untransfected Flp-In-TREx HEK293 cells and cells transfected with the WT receptor but uninduced exhibited a reduced proliferation (≈45% and ≈25%, respectively) as compared to those expressing the WT receptor (considered as 100%) (Figure 9A). Cells expressing the distinct mutant LPA_3_ receptors showed enhanced baseline proliferation (IL3, ≈1.7-fold; Ctail, ≈2.9-fold, and IL3/Ctail, ≈2-fold) (Figure 9A). As anticipated when the WT receptor-expressing cells were incubated with 10% fetal bovine serum, 1 µM LPA, 1 µM PMA, or 100 ng/mL EGF, proliferation markedly increased (Figure 9B). Despite the enhanced baseline proliferation, cells expressing the IL3 mutant receptor showed a similar pattern of enhanced proliferation in response to the agents tested (Figure 9B). In cells expressing the Ctail or the IL3/Ctail mutants, no significant proliferation increases in response to serum or LPA were observed, which could be related to the enhanced baseline proliferation observed with these cells. 

Similar experiments were carried out in cells incubated throughout the experiments (i.e., from induction to the end of the cell incubations) in the presence of 1 µM Ki16425, a well-known LPA_1,3_ antagonist. Ki16425 decreased baseline proliferation by ≈35% in cells expressing the WT receptors (Figure 9C,D). Interestingly, during these experiments, it was observed that (a) the baseline proliferation signal was no longer different among the cells expressing the distinct LPA_3_ receptors, (b) the actions of serum and LPA were essentially abolished, (c) the action of PMA was only marginally detected in the IL3/Ctail expressing cells, and (d) the ability of EGF to promote cell proliferation was robust and of a similar magnitude in the cells expressing the distinct LPA_3_ receptors but diminished when compared to that in the absence of inhibitor (from 7–8-fold increases to only ≈3-fold increases). 

**Figure 10:**
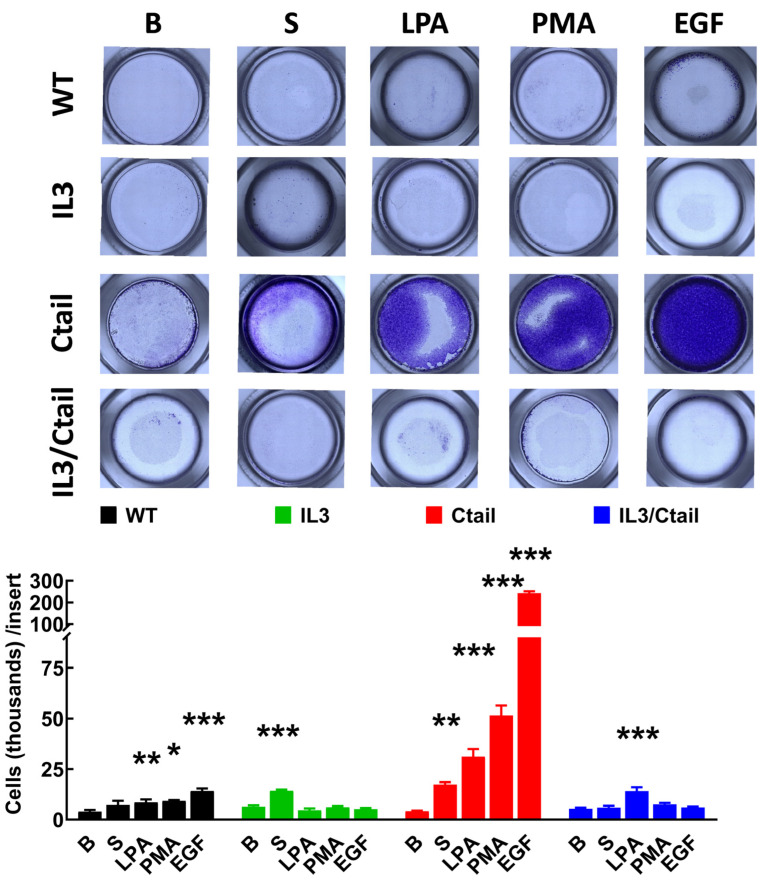
Migration of cells expressing the different LPA_3_ receptor constructs. Cells expressing the different LPA_3_ receptor constructs were plated in Boyden Chambers, and the attractants were tested; i.e., absence of attractant (B, baseline), 10% serum (S), 1 µM LPA, 1 µM PMA, or 100 ng/mL EGF. Data are expressed as thousands of cells/inserts. The means are plotted, and vertical lines indicate the SEM of 4 experiments performed on different days and cell cultures. *** *p* < 0.001, ** *p* < 0.01, and * *p* < 0.05vs. their respective baseline values. Above the graph, a photograph of the inserts of a representative experiment is shown.

**Figure 11:**
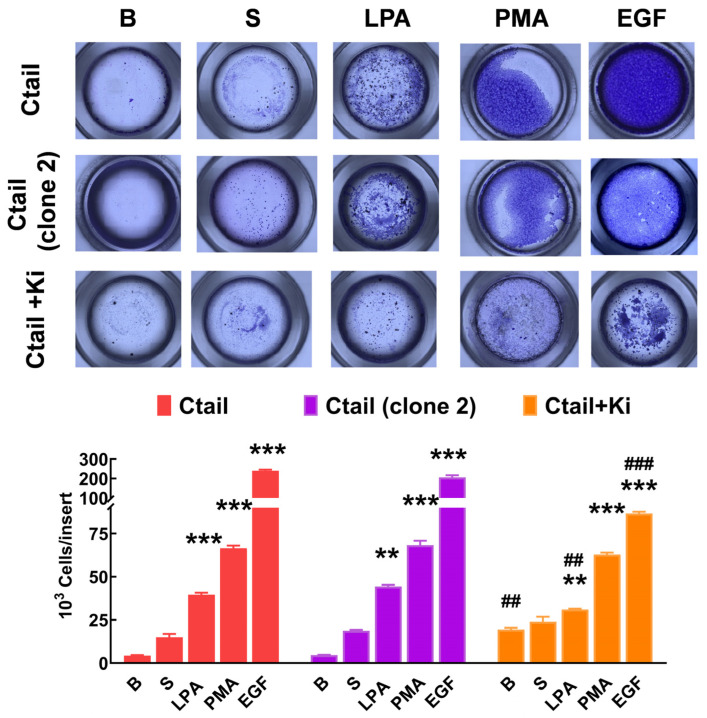
Migration of two different cell lines expressing the Ctail LPA_3_ receptor and effect of Ki16425. Cells were plated in Boyden chambers, and experiments were performed as described in Figure 11. In the left group of columns (red), the original cell line was employed, and in the middle group (purple), a cell line generated on a different day also expressing the same Ctail LPA_3_ receptor construct was used. The original cell line was employed in the right group of columns (orange), but the cells were incubated in media containing 1 µM Ki16425. The means are plotted, and vertical lines indicate the SEM of 3 experiments performed on different days and cell cultures. *** *p* < 0.001 and ** *p* < 0.01 vs. their respective baseline values. ## *p* < 0.01 and ### *p* < 0.001 vs. cells of the original group incubated without Ki16425.

Cell migration was studied with Boyden chambers using the different LPA_3_ receptors and serum, LPA, PMA, and EGF, as attractants (Figure 10). In cells expressing the WT LPA_3_ receptor, the different agents induced migration (although the effect of serum was small and did not reach statistical significance), EGF being the most effective. In cells expressing the IL3 mutant, only an effect of serum was observed, whereas, in cells expressing the IL3/Ctail mutant, only LPA increased migration (Figure 10). The figure also shows that in cells expressing the Ctail mutant, the effect of all attractants was much more prominent (as much as 10-fold in the case of EGF). We generated another line of cells expressing the Ctail mutant (named here Clone 2), and similar effects were observed, discarding the possibility that such an effect could be due to a peculiarity of the cell line (Figure 11). Incubation with Ki16425 increased by itself baseline migration. Additionally, this LPA_1-3_ inhibitor decreased migration in response to LPA and EGF in cells expressing the Ctail mutant (first clone) (Figure 11). 

A qualitative assessment of the functional repercussion of the distinct LPA_3_ mutations on the parameters studied is presented as Supplementary Table S1 at the end of the Supplementary Materials. 

## 3. Discussion

The present work employed a cellular model system in which LPA3 receptors were overexpressed; such overexpression limits the data’s extrapolation to physiological contexts. Unfortunately, no suitable cellular model system exists since most cells express different proportions of the distinct LPA receptors. Our show data confirm that the sites found phosphorylated in the mass spectrometry studies are those mainly involved in LPA_3_ phosphorylation, as evidenced by the fact that substitution by non-phosphorylatable amino acids essentially abolished baseline and stimulated receptor phosphorylation [15]. Receptor phosphorylation data using the partial substitutions (i.e., cells expressing either the IL3 or the Ctail mutants) indicate that both domains contribute to receptor phosphorylation. As indicated in the Section 2, a somewhat faster migration was observed in extracts from cells expressing mutants containing the Ctail substitutions (i.e., the Ctail and IL3/Ctail mutants). After this finding, the plasmids were sequenced again and found to be correct. Therefore, such a faster migration was not due to any sequence change. The changes in receptor phosphorylation themselves could likely be responsible for the faster receptor migration. It could also be attributed to differences in posttranslational receptor modifications, such as changes in glycosylation or proteolysis, which cannot be discarded. 

Changes in agonist-induced increases in calcium signaling were relatively small in the concentration–response curves and opposite to what we anticipated, based on similar experiments using the α_1B_-adrenergic receptors [28]. In cells expressing receptors with similar amino acid substitutions of this adrenergic receptor, we observed that cells expressing the IL3 or the Ctail mutants exhibited a shift to the left in the concentration–response curves, and this was even more pronounced in cells expressing the IL3/Ctail mutant [28]. The maximal response was markedly increased in these cells, and the return toward baseline was slower [28]. The data with the adrenergic receptor indicated that eliminating the phosphorylation sites allowed a fuller expression of the receptors’ apparent efficacy and potency in these cells (in other words, amino acid substitution eliminates some constraints). The present data employing cells expressing the LPA_3_ mutants were different and suggest that modification in the phosphorylation sites of this receptor might decrease the receptors’ affinity for LPA; the data also suggest that receptor phosphorylation might not be sufficient to desensitize the LPA_3_ receptor, which is likely related to the difficulties to observe LPA-induced LPA_3_ receptor desensitization, and the absence of effect on this parameter of GRK2 or GRK5 overexpression (submitted manuscript [15]). It should be mentioned that we did not observe, in the distinct mutants, any change in the baseline calcium concentration. However, we cannot discard the possibility of some endogenous or unrestrained receptor activity, because cells adapt rapidly to maintain, within small margins, their calcium concentration.

It was also unexpected that cells expressing the IL3/Ctail LPA_3_ mutant receptor exhibited a consistently high ERK 1/2 baseline signal. Such an increase was hard to define due to the intrinsic difficulties of the Western blotting technique, even controlling the exposure time very carefully. For this reason, we recurred to running in parallel cells and samples from cells expressing the WT receptor with those of the mutants, and data were normalized with the WT baseline signal (100%). The increased ERK phosphorylation of cells expressing the IL3/Ctail mutant was associated with enhanced baseline LPA_3_ receptor-β-arrestin interaction (FRET), baseline receptor internalization, and proliferation but not with cell migration. Interestingly, cells expressing the Ctail mutant showed a slight decrease in baseline ERK phosphorylation but a remarkable increase in attractant-induced migration.

The interaction of G protein-coupled receptors with β-arrestins seems to involve receptors’ acidic residues and phosphorylated sites [21,22]. Phosphorylation codes for β-arrestin recruitment by G protein-coupled receptors have been discovered [29], which allows finding putative β-arrestin binding sites in receptors. The LPA_3_ phosphorylation sites detected in the IL3 and Ctail (submitted manuscript [15]) were within putative interaction sites for β-arrestins [29]. Therefore, we anticipated that cells expressing the mutants, these receptors would interact poorly with β-arrestin. Surprisingly, the baseline FRET signal was increased in cells expressing the mutant receptors. However, the rapid LPA- or PMA-induced receptor-β-arrestin interaction (FRET) was markedly reduced or absent. Only a delayed response to LPA was observed in the cells expressing the Ctail mutant, and a delayed response to PMA was observed in cells expressing the IL3/Ctail mutant. 

Phosphorylation of G protein-coupled receptors promotes their interaction with β-arrestins, which plays a crucial role in receptor internalization [30,31,32,33,34]. However, there is also evidence of phosphorylation-independent receptor desensitization and internalization [35,36]. Our experiments showed that LPA- and PMA-induced receptor-β-arrestin 2 interaction was markedly reduced in the LPA_3_ mutants, paralleled with defective agonist- and PKC-induced internalization. It is worth mentioning that both the IL3 and Ctail mutants exhibited these defects, which is consistent but does not prove that phosphorylation sites in both domains participate in receptor-β-arrestin interaction and internalization. Additionally, a correlation seems to exist between the increased baseline FRET signal and the increased baseline intracellular density of fluorescence (receptors). It is possible that the amino acid substitutions performed might alter some steps of the receptor internalization/recycling to the plasma membrane, but this remains to be explored in the future. Despite this limitation, our data indicate the importance of IL3 and Ctail phosphorylation sites in agonist- and PKC-mediated internalization. 

Notably, the mutant receptors show slightly decreased sensitivity to LPA to increase intracellular calcium. Similarly, LPA-induced ERK 1/2 phosphorylation was relatively minor and generally maintained the same response pattern. The only change was the increased baseline signal in the IL3/Ctail mutant. These LPA effects were rapid, taking seconds (intracellular calcium) or minutes (ERK phosphorylation) to reach their maxima, and seem to be mediated through pertussis toxin-insensitive G proteins (likely G_q/11_) (submitted manuscript [15]). Substitution on the LPA_3_ receptor residues found phosphorylated does not decrease these effects closely associated with G protein signaling. 

Agonist effects that involve β-arrestin, such as LPA_3_-β-arrestin interaction and receptor internalization, were markedly decreased. ERK phosphorylation exhibits two phases: a rapid initial phase, mediated mainly through G protein-initiated pathways, and a second, more sustained phase that involves β-arrestins. Our experiments found no relationship between agonist-induced LPA_3_-β-arrestin interaction and ERK phosphorylation. Evidence indicates that agonists can activate ERK phosphorylation in the absence or presence of β-arrestins [37]. 

The mutant receptor-expressing cells had an increased baseline proliferation as evidenced in the MTT assay. It is noteworthy that the sole expression of the WT LPA3 receptor was able to promote baseline proliferation compared to those with the uninduced receptor. The increased baseline proliferation observed in cells expressing the mutant receptor was abolished by the LPA_1,3_ antagonist, i.e., under these conditions, the baselines of all the cells expressing the mutant receptor were identical to that of WT receptor-expressing cells.. The ability of Ki1425 to decrease baseline calcium and ERK 1/2 phosphorylation also supports the possibility of endogenous activity. It is possible that LPA traces present in the media or released by the cells might trigger receptor activity and that such activity could be less restrained in the mutant receptors. Endogenous LPA_3_ activity is likely, but formal demonstration requires pharmacological tools yet unavailable (such as a neutral or “classic” antagonist).

The cell migration studies with the LPA_3_ mutants also provided some surprising data. In contrast to what took place in proliferation, cells expressing the WT or the mutant receptors exhibit similar baseline migration. The response to the distinct attractants observed in cells expressing the Ctail mutant was unexpectedly intense. Ki16425 reduced, as expected, the effect of LPA, but surprisingly, also that of EGF. The reason for such a decrease is presently unknown. Cells expressing a mutated IL3 domain (i.e., IL3 and IL3/Ctail mutants) showed markedly decreased migration responses to PMA and EGF. In principle, the action of these agents could be independent of the LPA_3_ receptors. Therefore, the possibility that the modified IL3 domain might interfere with a fundamental step in the migration process should be considered.

Our data contribute to understanding receptor phosphorylation’s roles in the function and regulation of the LPA_3_ receptor. This work also provides a frame for future studies using structural and pharmacological approaches to clarify LPA_3_ receptor function and regulation intricacies.

## 4. Materials and Methods

### 4.1. Materials

1-Oleyl lysophosphatidic acid (LPA) was from Cayman Chemical Co. (Ann Arbor, MI, USA). Phorbol 12-myristate-13-acetate (PMA), and N-[5-[(4-Bromophenyl)methylene]-4, 5-dihydro-4-oxo-thiazolyl]-1-naphthalene-sulfonamide (Pitstop 2) were obtained from Sigma-Aldrich (St. Louis, MO, USA). MTT (3-[4,5-dimethylthiazol-2-yl]-2,5 diphenyl tetrazolium bromide), and DMSO (dimethyl sulfoxide) (1029521000) were purchased from Merck & Co. (Rahway, NJ, USA). Dulbecco’s modified Eagle’s medium, trypsin, Lipofectamine 2000, streptomycin, penicillin, amphotericin B, blasticidin, hygromycin B, doxycycline hyclate, and Fura-2 AM were purchased from Invitrogen-Life Technologies (Carlsbad, CA, USA). Fetal bovine serum was obtained from BioWest (Nuaillé, France). [^32^P]P_i_ (8500–9120 Ci/mmol) was obtained from American Radiolabeled Chemicals (St. Louis, MO, USA). Polyvinylidene difluoride membranes were purchased from Bio-Rad, Hercules, CA, USA, Lipofectamine 2000 (catalog number 11668-019, lot 1854318), and SuperSignal West Pico Chemiluminescence kits were purchased from Thermo Fisher Scientific (Waltham, MA, USA). Agarose-coupled protein A was obtained from Merck-Millipore (Burlington, MA, USA). Anti-phospho-ERK 1/2 (Thr202/Tyr204) (catalog number 9101S, Lot: 30) and anti-total ERK (p42/44) antibodies (catalog number 4695S, Lot: 21) were from Cell Signaling Technology (Danvers, MA, USA), monoclonal anti-GFP (green fluorescent protein) antibodies were from Clontech (JL-8; catalog number 632381, Lot: A8034133) (Mountain View, CA, USA). Rabbit polyclonal anti-GFP antisera were generated in our laboratory [38,39,40]. Primary antibodies were used at a dilution of 1:2000, whereas secondary antibody dilution was 1:10,000. The peroxidase affiniPure Goat anti-mouse IgG light-high chain antibody and other secondary antibodies were purchased from Zymed (Thermo Fisher Scientific; (Waltham, MA, USA)) or Jackson ImmunoResearch (West Grove, PA, USA). Human embryonic kidney (HEK) 293 cells were obtained from the American Type Culture Collection (HEK293; ATCC CRL-1573) (Manassas, VA, USA). Parental Flp-In T-Rex HEK293 cells and the plasmid, pOG44, were obtained from Invitrogen (Carlsbad, CA, USA). The plasmid for the expression of β-arrestin 2 tagged with the mCherry protein was generously provided by Dr. Adrian J. Butcher (University of Leicester, UK) [34]. We employed 96-well Cell Culture Plates from SPL Life Sciences (Pocheon-si, Republic of Korea) for the proliferation studies and Transwell Permeable Support 6.5 mm Insert, 24 Well Plate 8.0 µm PET Membrane Polystyrene from Costar-Corning (Lot 19223024) (Glendale, CA, USA) to study cell migration.

### 4.2. Cell Lines

The LPA_3_ receptor sequence was fused at the carboxyl terminus (Ctail) with the GFP and cloned into the pCDNA5/FRT/TO plasmid (Bioinnovatise, Inc., Rockville, MD, USA) to employ the inducible Flp-In TREx expression system and this sequence, was employed as a template to generate substitutions of the sites found phosphorylated for non-phosphorylatable amino acids. The Flp-In-TREx cellular system allowed tetracycline-induced receptor expression [41]. Mutant plasmids were commercially obtained (Bioinnovatise, Inc., Rockville, MD, USA) and confirmed by sequencing. These include: (a) a mutant in which the sites found phosphorylated in the third intracellular loop were modified (S221A, T224A, S225A, and S229A), named for brevity IL3 mutant in this manuscript; (b) a mutant substituting sites observed phosphorylated in the carboxyl terminus (S321A, S325A, S331A, T333V, S335A, Y337F, and S343A), referred to as Ctail mutant; and (c) a mutant in which all the previously indicated sites were substituted in both domains, named IL3/Ctail mutant (a cartoon displaying LPA_3_ WT and mutant receptors is depicted in Supplementary Figure S1). Parenteral cells Flp-In-TREx HEK293, co-transfected with the pOG44. Cells were subjected to selection for one month using Dulbecco’s modified Eagle’s medium supplemented with 10% of fetal bovine serum 100 μg/mL streptomycin, 100 U/mL penicillin, and 0.25 μg/mL amphotericin B, 10 µg/mL blasticidin and 100 µg/mL hygromycin B. Surviving colonies were collected and lines expressing the LPA_3_ mutant receptors were selected based on their receptor expression in response to induction with 10 µg/mL doxycycline hyclate for 12 h, evidenced using fluorescence microscopy (representative images are shown in Supplementary Figure S5), and their ability to increase the concentration of intracellular calcium in response to LPA.

### 4.3. Receptor Phosphorylation

Cells expressing the LPA_3_ receptor mutants were incubated for 1 h in phosphate-free Dulbecco’s Modified Eagle’s media, followed by incubation for 3 h in the same media but supplemented with 50 μCi/mL [^32^P]P_i_. After these incubations, cells were treated with the agents for the time indicated, washed with ice-cold phosphate-buffered saline, and solubilized for 1 h in the lysis buffer [26]. The extracts were centrifuged for 15 min at 13,000 rpm, and the supernatants were incubated overnight with protein A-agarose and the anti-GFP antiserum generated in our laboratory. The next day, samples were washed five times, and the pellets were denaturalized with sample buffer [42]. Proteins were separated using 10% SDS-polyacrylamide gel electrophoresis, electrotransferred onto nitrocellulose membranes, and exposed for 24 h. The amount of phosphorylated receptor was assessed by PhosphorImager analysis using the ImageQuant program version 5.0. The same membranes were subjected to Western blotting (loading control) utilizing a commercial monoclonal anti-GFP antibody.

### 4.4. Intracellular Calcium Concentration

Intracellular calcium was determined as described [15,26]. In brief, cells were loaded with 2.5 µM Fura-2 AM for 1 h at 37 °C. After the incubation, the cells were washed 2 times with buffer to eliminate unincorporated dye, and determinations were carried out in suspended cells with an AMINCO-Bowman Series 2 luminescence spectrometer, employing 340 and 380 nm excitation wavelengths and an emission wavelength of 510 nm, with a chopper interval of 0.5 s. Intracellular calcium levels were calculated as described by Grynkiewicz et al. [43].

### 4.5. ERK 1/2 Phosphorylation

The cells were serum-starved for 4 h. The cells in Petri dishes were incubated with the agents and for the times indicated. After this incubation, the medium was removed, and the cells were washed with ice-cold phosphate-buffered saline; lysis buffer was added, and the lysates were maintained for 1 h on a bed of ice; lysates were centrifuged, and the supernatants were recovered and denatured using Laemmli sample buffer [42]. Samples were separated using polyacrylamide-SDS gel electrophoresis, and samples were electrotransferred onto polyvinylidene difluoride membranes, and Western blotting was performed. The expression of the levels of pERK1/2 and total ERK were determined on the same membrane. The baseline values were considered 100% for normalization.

### 4.6. LPA_3_ Receptor-β-Arrestin 2 Interaction

The interaction of the distinct receptor mutants with β-arrestin 2 was determined using Föster Resonance Energy Transfer (FRET) analysis, as described previously [15]. These cells coexpressed the WT LPA_3_ receptor, or the mutants, tagged with the GFP and β-arrestin 2 tagged with the mCherry fluorescent protein. LPA_3_-β-arrestin interaction was analyzed using an FV10i Olympus microscope with an automated laser spectral scan (Olympus, Hamburg, Germany). The GFP was excited at 488 nm, and the emitted fluorescence was detected at 510 nm, whereas mCherry was excited at 580 nm and emitted fluorescence was detected at 610 nm; this was routinely performed, and images were obtained to check the expression of these proteins. For FRET channel analysis, GFP (but not mCherry) was excited, and fluorescence was detected at 510 nm; such fluorescence indicated that the proximity among the fluorescent proteins was enough to allow energy transfer (i.e., 1–10 nm) [44]. The FRET index was quantified using ImageJ software (version 1.49v), which removes bleed-through and false FRET. The images were analyzed using 8 bits that permit pixel-by-pixel supervised computational FRET index analysis. The average FRET index obtained with the vehicle (time 0 min) was normalized as 100%. Individual cells (not clusters) expressing both fluorescent proteins were randomly selected; 7–8 cells were analyzed for each experimental condition in all the experiments.

### 4.7. Receptor Internalization

These experiments were performed as described previously [15,28,44]. Cells were seeded at low density on glass-bottomed Petri dishes for 12 h. Receptor expression was induced for 12 h, and cells were serum-fasted for 1h before stimulation with the agents and for the times indicated. After stimulation, the cells were washed twice with phosphate-buffered saline and immediately fixed. The images were obtained using a Fluoview Confocal model FV10i microscope (Olympus, Hamburg, Germany); GFP was excited at 480 nm and emitted fluorescence registered at 515–540 nm. The plasma membrane was delineated using differential interference contrast images to determine receptor internalization. Each cell’s intracellular fluorescence (i.e., excluding the plasma membrane) was quantified as “integrated density”, employing the ImageJ software [45,46]. The procedure is described in detail for “Corrected total cell fluorescence” (in [45] and in The Open Lab Book (https://theolb.readthedocs.io/en/latest/imaging/measuring-cell-fluorescence-using-imagej.html; accessed on 20 January 2024)). Usually, 10–14 images were taken from 3 or 4 cultures obtained on different days for each condition.

### 4.8. Proliferation

Cells were seeded in 96-well plates at ≈10,000 cells/well density, and receptor expression was induced for 12 h. After induction, cells were treated with the agents indicated for 16 h (at this time, more apparent effects were observed). After this treatment, 0.25 μg/μL MTT was added, and the mixture was incubated at 37 °C for 4 h; adding a DMSO/10% SDS lysis solution dissolved the formazan salts formed [27]. The plates were shaken at 30 min at room temperature and absorbance was determined at 550 nm using a Citation 3 Image BioteK reader, Bio Tek, Agilent, Santa Clara, CA, USA). Samples processed similarly, but without cells, were used as a blank. Proliferation was calculated by the difference between the optical density of treated cells and that of untreated cells, employing the following formula: % proliferation = (OD treatment − OD blank)/(OD vehicle − OD blank) × 100% [27].

### 4.9. Migration Assays

Cells transfected to express the WT or the LPA_3_ receptor mutants were seeded in the transwell plate upper compartments (inserts) of Boyden chambers at a density of ≈10,000 cells/well and were incubated to allow attachment for 12 h; after this period, receptor expression was induced by addition of doxycycline (final 10 µg/mL), and incubation was continued for an additional 12 h and the medium was aspirated from the inserts. Immediately, serum-free media containing the different stimuli (vehicle, 10% fetal bovine serum, 1 µM LPA, 1 µM PMA, or 100 ng/mL EGF) were added to the lower compartments, both compartments were assembled, and plates were incubated for 48 h in the cell incubator chamber (37 °C, humidity, and 5% CO_2_/95% O_2_). After this period, the inserts are washed twice with phosphate-buffer saline. Cells in the upper part of the insert were removed with a cotton swab to subsequently fix the cells in the lower part of the insert for 5 min with 4% para-formaldehyde; the samples were washed twice with saline phosphate buffer, and samples permeabilized for 15 min with 100% methanol. Finally, the samples in the inserts were stained by incubation for 20 min with a 1% crystal violet solution and washed with phosphate buffer saline 2 times. The stained inserts were photographed using a stereoscopic microscope Axio Zoom V 1.6. Quantification was performed using the ImageJ program (particle analyzer tool version 1.54) [45,46]. 

### 4.10. Statistical Analyses

Statistical analyses between comparable groups were performed using ordinary one-way ANOVA with the Bonferroni post-test, employing the software included in the GraphPad Prism program (version 10.2.2). A *p*-value < 0.05 was considered statistically significant.

## Data Availability

The data presented in this study are available upon request from the corresponding author.

## Supplementary Materials

The supporting information can be downloaded at https://www.mdpi.com/article/10.3390/ijms25105508/s1.

## References

[1] Kihara Y., Maceyka M., Spiegel S., Chun J. (2014). Lysophospholipid receptor nomenclature review: IUPHAR Review 8. Br. J. Pharmacol..

[2] Choi J.W., Herr D.R., Noguchi K., Yung Y.C., Lee C.W., Mutoh T., Lin M.E., Teo S.T., Park K.E., Mosley A.N. (2010). LPA receptors: Subtypes and biological actions. Annu. Rev. Pharmacol. Toxicol..

[3] Geraldo L.H.M., Spohr T., Amaral R.F.D., Fonseca A., Garcia C., Mendes F.A., Freitas C., dosSantos M.F., Lima F.R.S. (2021). Role of lysophosphatidic acid and its receptors in health and disease: Novel therapeutic strategies. Signal Transduct. Target. Ther..

[4] Chan L.C., Peters W., Xu Y., Chun J., Farese R.V., Cases S. (2007). LPA3 receptor mediates chemotaxis of immature murine dendritic cells to unsaturated lysophosphatidic acid (LPA). J. Leukoc. Biol..

[5] Tanabe E., Kitayoshi M., Yoshikawa K., Shibata A., Honoki K., Fukushima N., Tsujiuchi T. (2012). Loss of lysophosphatidic acid receptor-3 suppresses cell migration activity of human sarcoma cells. J. Recept. Signal Transduct. Res..

[6] Goldsmith Z.G., Ha J.H., Jayaraman M., Dhanasekaran D.N. (2011). Lysophosphatidic Acid Stimulates the Proliferation of Ovarian Cancer Cells via the gep Proto-Oncogene Galpha(12). Genes Cancer.

[7] Chiang J.C., Chen W.M., Lin K.H., Hsia K., Ho Y.H., Lin Y.C., Shen T.L., Lu J.H., Chen S.K., Yao C.L. (2021). Lysophosphatidic acid receptors 2 and 3 regulate erythropoiesis at different hematopoietic stages. Biochim. Biophys. Acta Mol. Cell Biol. Lipids.

[8] Ye X., Hama K., Contos J.J., Anliker B., Inoue A., Skinner M.K., Suzuki H., Amano T., Kennedy G., Arai H. (2005). LPA3-mediated lysophosphatidic acid signalling in embryo implantation and spacing. Nature.

[9] Lai S.L., Yao W.L., Tsao K.C., Houben A.J., Albers H.M., Ovaa H., Moolenaar W.H., Lee S.J. (2012). Autotaxin/Lpar3 signaling regulates Kupffer’s vesicle formation and left-right asymmetry in zebrafish. Development.

[10] Balijepalli P., Sitton C.C., Meier K.E. (2021). Lysophosphatidic Acid Signaling in Cancer Cells: What Makes LPA So Special?. Cells.

[11] Zhao P., Yun Q., Li A., Li R., Yan Y., Wang Y., Sun H., Damirin A. (2022). LPA3 is a precise therapeutic target and potential biomarker for ovarian cancer. Med. Oncol..

[12] Yung Y.C., Stoddard N.C., Chun J. (2014). LPA receptor signaling: Pharmacology, physiology, and pathophysiology. J. Lipid Res..

[13] Solís K.H., Romero-Ávila M.T., Guzmán-Silva A., García-Sáinz J.A. (2021). The LPA(3) Receptor: Regulation and Activation of Signaling Pathways. Int. J. Mol. Sci..

[14] Meduri B., Pujar G.V., Durai Ananda Kumar T., Akshatha H.S., Sethu A.K., Singh M., Kanagarla A., Mathew B. (2021). Lysophosphatidic acid (LPA) receptor modulators: Structural features and recent development. Eur. J. Med. Chem..

[15] Solís K.H., Romero-Ávila M.T., Rincón-Heredia R., García-Sáinz J.A. (2024). LPA3 Receptor Phosphorylation Sites: Roles in Signaling and Internalization. Preprints.

[16] Vázquez-Prado J., Casas-González P., García-Sáinz J.A. (2003). G protein-coupled receptor cross-talk: Pivotal roles of protein phosphorylation and protein-protein interactions. Cell. Signal..

[17] Tobin A.B., Butcher A.J., Kong K.C. (2008). Location, location, location...site-specific GPCR phosphorylation offers a mechanism for cell-type-specific signalling. Trends Pharmacol. Sci..

[18] Prihandoko R., Bradley S.J., Tobin A.B., Butcher A.J. (2015). Determination of GPCR Phosphorylation Status: Establishing a Phosphorylation Barcode. Curr. Protoc. Pharmacol..

[19] Martínez-Morales J.C., Romero-Ávila M.T., Reyes-Cruz G., García-Sáinz J.A. (2022). Roles of Receptor Phosphorylation and Rab Proteins in G Protein-Coupled Receptor Function and Trafficking. Mol. Pharmacol..

[20] Gurevich V.V., Gurevich E.V. (2018). Arrestins and G proteins in cellular signaling: The coin has two sides. Sci. Signal.

[21] Gurevich V.V., Gurevich E.V. (2019). Plethora of functions packed into 45 kDa arrestins: Biological implications and possible therapeutic strategies. Cell Mol. Life Sci..

[22] Gurevich V.V., Gurevich E.V. (2019). GPCR Signaling Regulation: The Role of GRKs and Arrestins. Front. Pharmacol..

[23] Tobin A.B. (2008). G-protein-coupled receptor phosphorylation: Where, when and by whom. Br. J. Pharmacol..

[24] Alharbi A.G., Tobin A.B., Milligan G. (2022). How Arrestins and GRKs Regulate the Function of Long Chain Fatty Acid Receptors. Int. J. Mol. Sci..

[25] Martínez-Morales J.C., Solís K.H., Romero-Ávila M.T., Reyes-Cruz G., García-Sáinz J.A. (2022). Cell Trafficking and Function of G Protein-coupled Receptors. Arch. Med. Res..

[26] Alcántara-Hernández R., Hernández-Méndez A., Campos-Martínez G.A., Meizoso-Huesca A., García-Sáinz J.A. (2015). Phosphorylation and Internalization of Lysophosphatidic Acid Receptors LPA1, LPA2, and LPA3. PLoS ONE.

[27] Nga N.T.H., Ngoc T.T.B., Trinh N.T.M., Thuoc T.L., Thao D.T.P. (2020). Optimization and application of MTT assay in determining density of suspension cells. Anal. Biochem..

[28] Hernandez-Espinosa D.A., Alcantara-Hernandez R., Solis K.H., Garcia-Sainz J.A. (2023). Roles of the alpha(1B)-Adrenergic Receptor Phosphorylation Domains in Signaling and Internalization. Int. J. Mol. Sci..

[29] Zhou X.E., He Y., de Waal P.W., Gao X., Kang Y., Van Eps N., Yin Y., Pal K., Goswami D., White T.A. (2017). Identification of Phosphorylation Codes for Arrestin Recruitment by G Protein-Coupled Receptors. Cell.

[30] Fessart D., Simaan M., Laporte S.A. (2005). c-Src regulates clathrin adapter protein 2 interaction with beta-arrestin and the angiotensin II type 1 receptor during clathrin- mediated internalization. Mol. Endocrinol..

[31] Qian J., Wu C., Chen X., Li X., Ying G., Jin L., Ma Q., Li G., Shi Y., Zhang G. (2014). Differential requirements of arrestin-3 and clathrin for ligand-dependent and -independent internalization of human G protein-coupled receptor 40. Cell. Signal.

[32] Laporte S.A., Oakley R.H., Zhang J., Holt J.A., Ferguson S.S., Caron M.G., Barak L.S. (1999). The beta2-adrenergic receptor/betaarrestin complex recruits the clathrin adaptor AP-2 during endocytosis. Proc. Natl. Acad. Sci. USA.

[33] Claing A., Laporte S.A., Caron M.G., Lefkowitz R.J. (2002). Endocytosis of G protein-coupled receptors: Roles of G protein-coupled receptor kinases and beta-arrestin proteins. Prog. Neurobiol..

[34] Laporte S.A., Oakley R.H., Holt J.A., Barak L.S., Caron M.G. (2000). The interaction of beta-arrestin with the AP-2 adaptor is required for the clustering of beta 2-adrenergic receptor into clathrin-coated pits. J. Biol. Chem..

[35] Jala V.R., Shao W.H., Haribabu B. (2005). Phosphorylation-independent beta-arrestin translocation and internalization of leukotriene B4 receptors. J. Biol. Chem..

[36] Ferguson S.S. (2007). Phosphorylation-independent attenuation of GPCR signalling. Trends Pharmacol. Sci..

[37] Alvarez-Curto E., Inoue A., Jenkins L., Raihan S.Z., Prihandoko R., Tobin A.B., Milligan G. (2016). Targeted Elimination of G Proteins and Arrestins Defines Their Specific Contributions to Both Intensity and Duration of G Protein-coupled Receptor Signaling. J. Biol. Chem..

[38] Colín-Santana C.C., Avendaño-Vázquez S.E., Alcántara-Hernández R., García-Sáinz J.A. (2011). EGF and angiotensin II modulate lysophosphatidic acid LPA(1) receptor function and phosphorylation state. Biochim. Biophys. Acta.

[39] Hernández-Méndez A., Alcántara-Hernández R., Acosta-Cervantes G.C., Martínez-Ortiz J., Avendaño-Vázquez S.E., García-Sáinz J.A. (2014). Conventional protein kinase C isoforms mediate phorbol ester-induced lysophosphatidic acid LPA1 receptor phosphorylation. Eur. J. Pharmacol..

[40] Hernández-Méndez A., Alcántara-Hernández R., García-Sáinz J.A. (2014). Lysophosphatidic Acid LPA 1-3 receptors: Signaling, regulation and in silico analysis of their putative phosphorylation sites. Recept. Clin. Invest..

[41] Ward R.J., Alvarez-Curto E., Milligan G. (2011). Using the Flp-In T-Rex system to regulate GPCR expression. Methods Mol. Biol..

[42] Laemmli U.K. (1970). Cleavage of structural proteins during the assembly of the head of bacteriophage T4. Nature.

[43] Grynkiewicz G., Poenie M., Tsien R.Y. (1985). A new generation of Ca2+ indicators with greatly improved fluorescence properties. J. Biol. Chem..

[44] Hartig S.M. (2013). Basic image analysis and manipulation in ImageJ. Curr. Protoc. Mol. Biol..

[45] Schindelin J., Arganda-Carreras I., Frise E., Kaynig V., Longair M., Pietzsch T., Preibisch S., Rueden C., Saalfeld S., Schmid B. (2012). Fiji: An open-source platform for biological-image analysis. Nat. Methods.

[46] Rasband W.S. ImageJ. In National Institutes of Health. 1997–2004. http://rsb.info.nih.gov/ij/.

