# Supplementary material for: LPA3 Receptor Phosphorylation Sites: Roles in Signaling and Internalization
Source: Int J Mol Sci. 2024 May 18;25(10):5508. doi: 10.3390/ijms25105508 (PMC11122405; doi:10.3390/ijms25105508)
Supplement: Supplementary file 1 [file ijms-25-05508-s001.zip › ijms-2975908-supplementary.pdf]

Supplementary Materials

Supplementary Fig. S1. Cartoon representing the LPA$_3$ mutants employed indicating the substitutions performed**.**

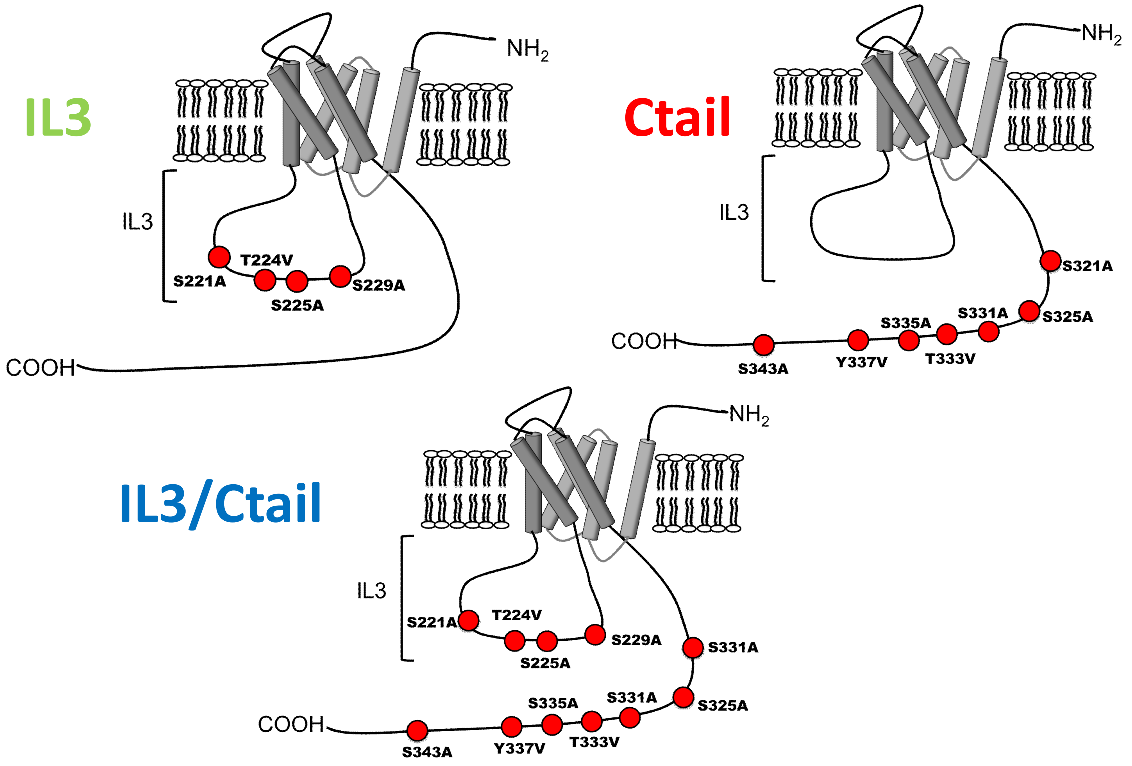

Supplementary Fig. S2. Effect of Ki16425 on intracellular calcium in cells expressing WT or mutant LPA_3 receptors. Representative calcium tracing of the effect of the addition (arrow) of 1 µM Ki16425 (Ki) in cells expressing WT (panel A), IL3 (panel B), Ctail (panel C) or IL3/Ctail (panel D) LPA_3 receptors. In panel E the decreases in intracellular calcium are presented. The means are plotted, and vertical lines indicate the SEM of 4-5 experiments performed on different days using distinct cell cultures.***$p < 0.001$ vs. baseline; ** $p < 0.01$ vs. baseline.

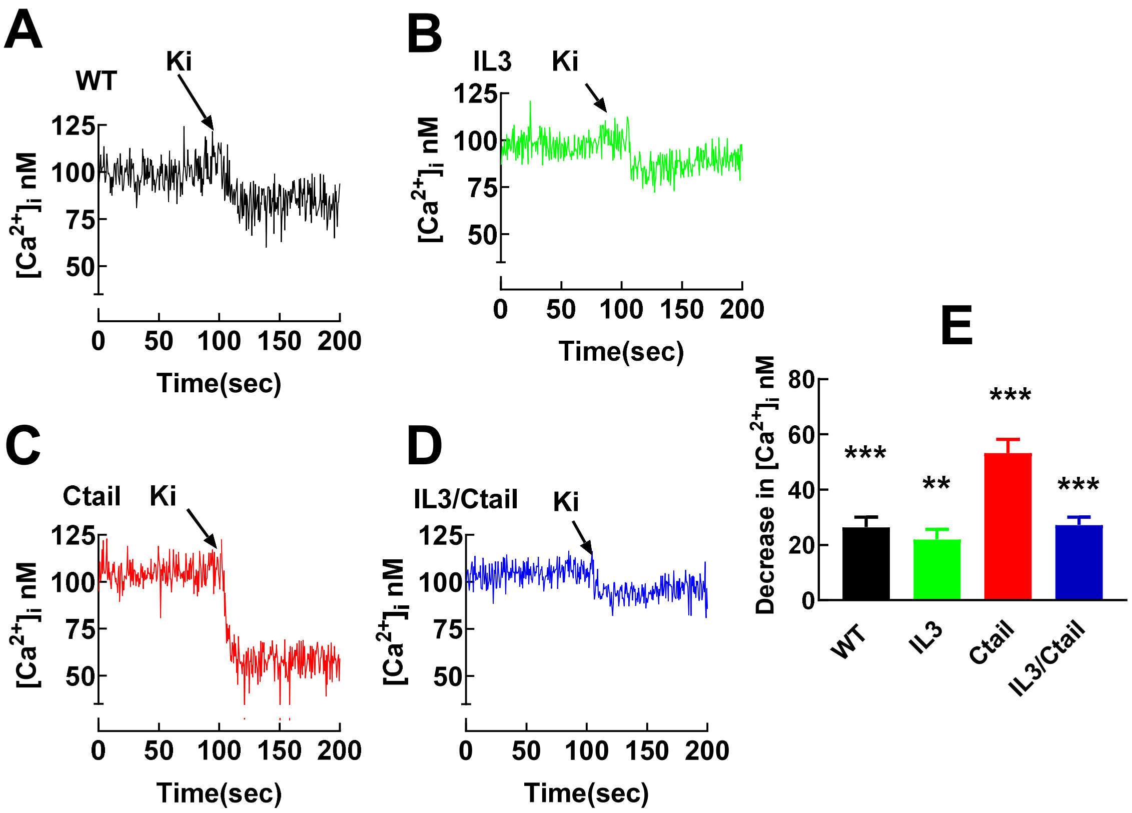

Supplementary Fig. S3. Time-course of LPA- and PMA-induced ERK 1/2 phosphorylation in cells expressing WT or mutant LPA_3 receptors. Experiments were run in parallel with cells expressing WT and mutant receptors to compare the effects properly. Cells were stimulated with 1 μM LPA (Panel A) or 1 μM PMA (Panel B) for 0, 2, and 60 min. Data are presented as the percentage of the baseline observed in cells expressing the WT receptor. The means are plotted, and vertical lines indicate the SEM of 5-7 experiments performed on different days using distinct cell cultures. Representative Western blots are presented above the figure.

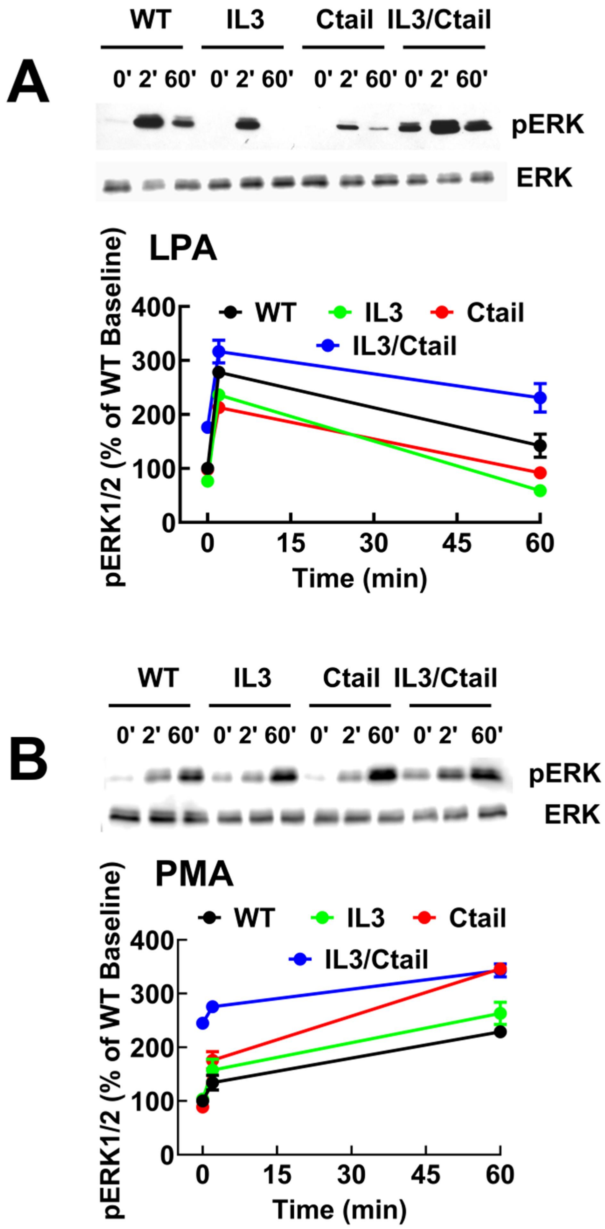

Supplementary Fig. S4. Effect of Ki16425 on ERK 1/2 phosphorylation in cells expressing WT or mutant LPA_3 receptors. Cell expressing the WT LPA_3 receptors (panel A) or the IL3 (panel B), Ctail (panel C) or il3/Ctail (panel D) mutants were incubated for 15 min in the absence or presence of 1 µM Ki16425 (+Ki) and then challenged by vehicle (B), 100 nM LPA or 100 ng/ml EGF. Data are presented as percentage of the baseline observed with cells expressing the same receptor construct. The means are plotted, and vertical lines indicate the SEM of 6 experiments performed on different days using distinct cell cultures.***$p < 0.001$ absence vs. presence of inhibitor.

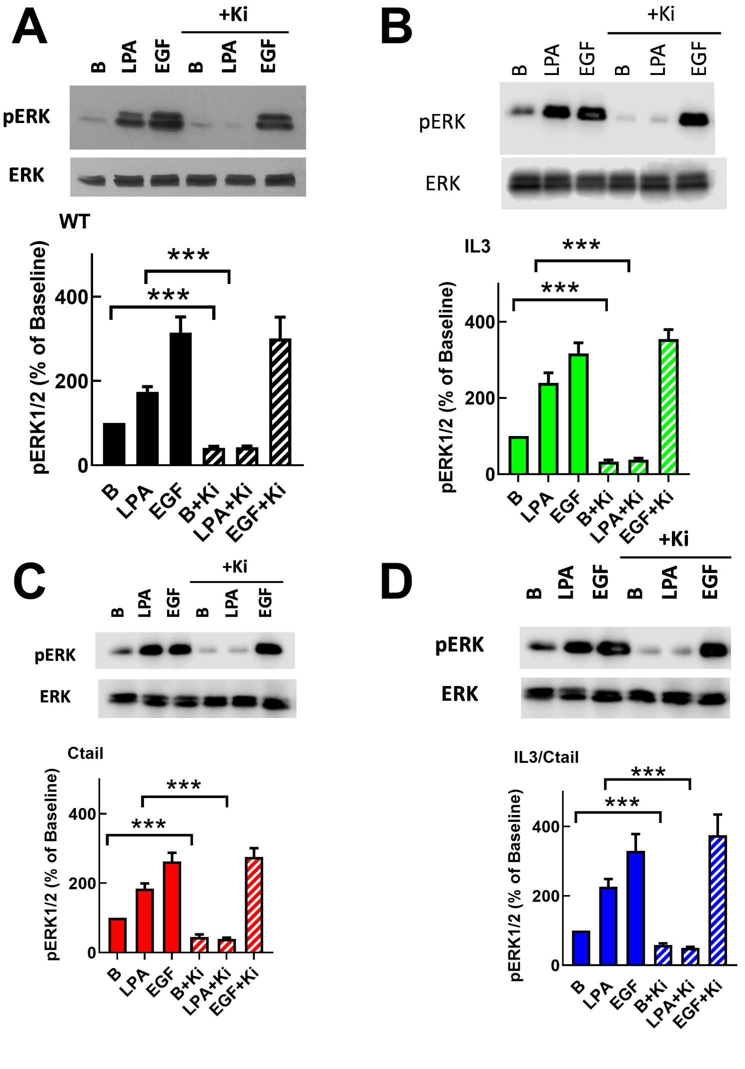

Supplementary Fig. S5. The images in the left column are the differential interference contrast, and those in the right column the fluorescent images of cells expressing the different LPA$_3$ receptor constructs used. Bars. 10 µm.

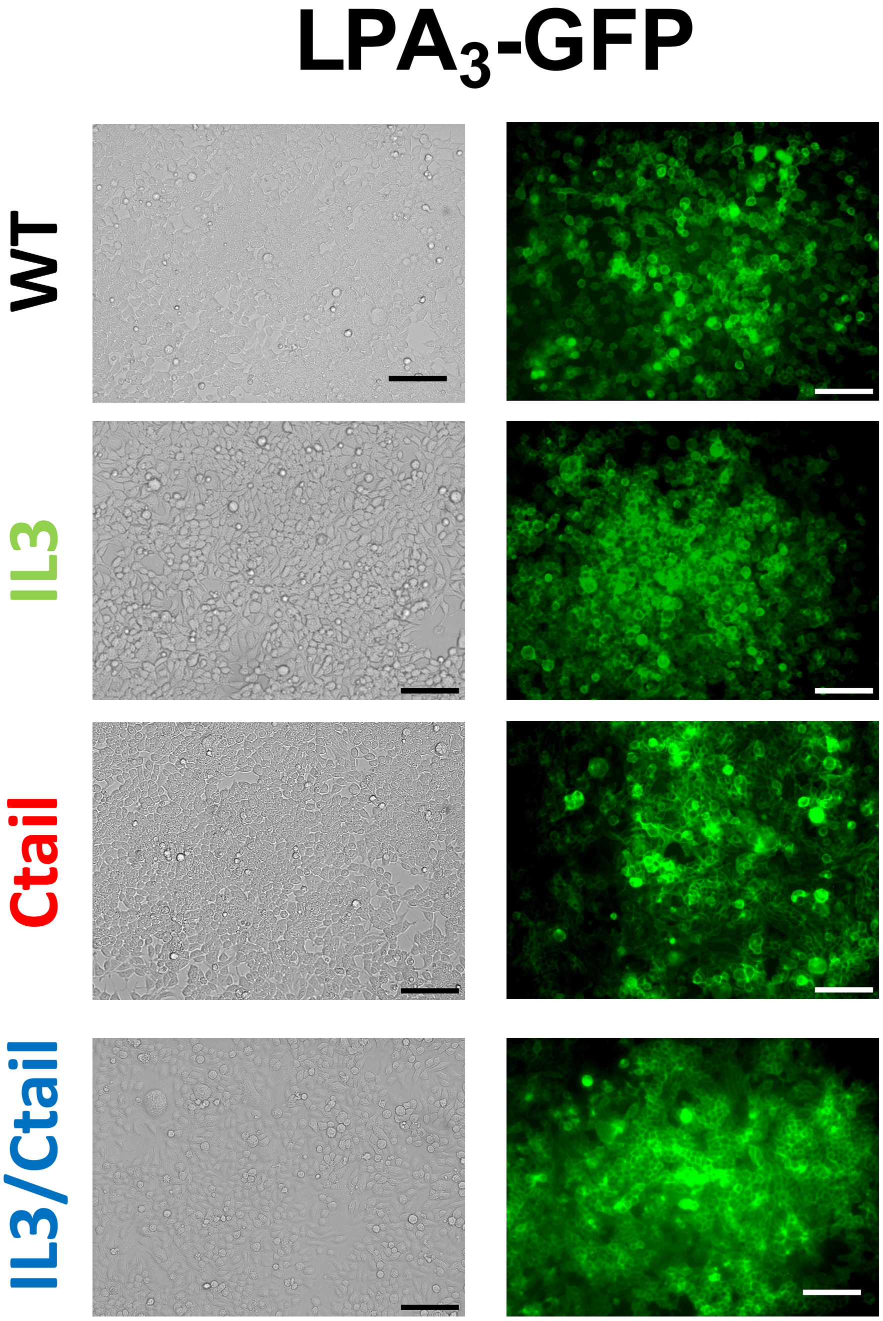

Supplementary Table S1

Qualitative assessment of the functional repercussion of the distinct LPA_3 mutations on the parameters studied as compared to the wild-type. Decreased (↓) and increased (↑).

Parameter	IL3	Ctail	IL3/ Ctail
**Receptor Phosphorylation**	**Baseline ↓↓**<br>**LPA ↓**<br>**PMA ↓**	**Baseline ↓**<br>**LPA ↓**<br>**PMA ↓**	**Baseline ↓↓↓**<br>**LPA ↓↓↓**<br>**PMA ↓↓↓**
**LPA action on $[Ca^{2+}]$ i**	**EC_{50} =**<br>**Max =**	**EC_{50} =/↑**<br>**Max =**	**App EC_{50} ↑**<br>**Max =**
**βarr FRET**	**Baseline =**<br>**LPA**	**Baseline ↑**<br>**LPA ↓delayed**	**Baseline ↑↑↑**<br>**LPA ↓↓↓**
**ERK 1/2 phosphorylation**	**Baseline =**<br>**LPA =/↓**<br>**PMA =**	**Baseline =**<br>**LPA ↓↓**<br>**PMA =/↑**	**Baseline ↑↑↑**<br>**LPA =/↑**<br>**PMA =**
**Receptor internalization**	**Baseline ↑**<br>**LPA ↓**<br>**PMA ↓**	**Baseline ↑↑**<br>**LPA ↓↓↓**<br>**PMA ↓↓↓**	**Baseline ↑↑**<br>**LPA ↓↓↓**<br>**PMA ↓↓↓**
**Proliferation (MTT)**	**Baseline ↑↑**<br>**Serum =**<br>**LPA =**	**Baseline ↑↑↑**<br>**Serum ↓**<br>**LPA ↓**	**Baseline ↑↑↑**<br>**Serum ↓**<br>**LPA =/↓**

	**PMA =**<br><br>**EGF =**	**PMA =**<br><br>**EGF =**	**PMA =**<br><br>**EGF =**
**Migration (Boyden chambers)**	**Baseline =**<br><br>**Serum ↑↑**<br><br>**LPA ↓↓↓**<br><br>**PMA ↓↓↓**<br><br>**EGF ↓↓↓**	**Baseline =**<br><br>**Serum ↑↑↑**<br><br>**LPA ↑↑↑**<br><br>**PMA ↑↑↑**<br><br>**EGF ↑↑↑**	**Baseline =**<br><br>**Serum ↓**<br><br>**LPA ↑↑**<br><br>**PMA ↓**<br><br>**EGF ↓↓**
